# Berbamine sensitizes hepatocellular carcinoma to chemotherapy by inhibiting autophagy *via* modulating SIRT1-mediated acetylation

**DOI:** 10.3389/fphar.2026.1763828

**Published:** 2026-05-14

**Authors:** Qin Peng, Ling Xiao, Xinhui Huang, Ziyuan Huang, Guangxian Zhang, Jihong Zhou, Yuhui Tan

**Affiliations:** 1 Shenzhen Bao’an Traditional Chinese Medicine Hospital, Guangzhou University of Chinese Medicine, Shenzhen, China; 2 Research Center of Integrative Medicine, School of Basic Medical Sciences, Guangzhou University of Chinese Medicine, Guangzhou, China; 3 Department of Biochemistry, Guangzhou University of Chinese Medicine, Guangzhou, China; 4 School of Life Sciences and Technology, Tongji University, Shanghai, China

**Keywords:** autophagy, berbamine, chemosensitivity, hepatocellular carcinoma, SIRT1, TFEB

## Abstract

Chemoresistance driven by pro-survival autophagy remains a major obstacle in hepatocellular carcinoma (HCC) treatment. Berbamine (BBM), a natural alkaloid with a favorable clinical safety profile, shows potential as an autophagy inhibitor, yet its precise mechanism in HCC remains unclear. Using CCK-8, colony formation, and apoptosis assays, we first demonstrated that BBM synergistically enhanced the efficacy of multiple chemotherapeutic agents (5-FU, Sorafenib, Paclitaxel) against HCC cells *in vitro*. This synergistic effect was confirmed in an H22 xenograft mouse model *in vivo*. To investigate the mechanism, we monitored autophagic flux and lysosomal function. Western blot and immunofluorescence analyses revealed that BBM treatment led to the concurrent accumulation of LC3-II and p62, indicating a blockade of late-stage autophagic flux. Further experiments, including LysoTracker staining and assessment of lysosomal protease levels, showed that BBM impaired both autophagosome-lysosome fusion and lysosomal acidification. Mechanistically, we found that BBM downregulated SIRT1 protein expression and reduced the intracellular NAD^+^/NADH ratio, thereby inhibiting SIRT1 deacetylase activity. This suppression impaired the nuclear translocation and function of the key autophagy transcription factor TFEB, leading to decreased levels of its downstream targets RAB7, CTSB, and CTSD. Crucially, rescue experiments using specific agonists revealed that SIRT1 activation completely reversed all BBM-induced effects, including autophagic flux blockade and downstream protein suppression, whereas TFEB activation only partially rescued the expression of RAB7, CTSB, and CTSD without restoring autophagic flux. This establishes SIRT1 as the primary upstream regulator in this pathway. Our study identifies BBM as a novel autophagy inhibitor that targets the SIRT1-TFEB axis to disrupt autolysosomal fusion and degradation, and nominates it as a promising combinational agent to overcome chemoresistance in HCC.

## 1 Introduction

Hepatocellular carcinoma (HCC) is the most prevalent cancer and the third leading cause of cancer-related deaths worldwide (Wei et al., 2022). It is characterized by poor prognosis and low 5-year survival rates. Pharmacological treatments have been proven effective in combating liver cancer. However, only a small fraction of HCC patients exhibit a sensitive response, and advanced-stage patients frequently develop drug resistance. Multidrug resistance in HCC poses a significant clinical challenge in its treatment (Wu et al., 2021; Xue et al., 2024).

Recent studies have shown that autophagy is closely linked to the development and maintenance of drug resistance (Elleithi et al., 2024). Autophagy is a conserved catabolic process wherein cellular components are sequestered within autophagosomes and delivered to lysosomes for degradation, thereby recycling nutrients and maintaining cellular homeostasis under stress (Galluzzi and Green, 2019). In cancer, this process can promote tumor cell survival against therapeutic insults, including chemotherapy (Qin et al., 2023). Consequently, inhibiting autophagy has emerged as a promising strategy to re-sensitize cancer cells, with clinical trials ongoing for agents like chloroquine (CQ) and hydroxychloroquine (HCQ) (Jain et al., 2023). However, the clinical translation of these inhibitors is often hampered by off-target toxicities at effective doses, underscoring the need for safer alternatives.

The regulation of autophagy involves intricate layers of post-translational modifications, among which acetylation plays a pivotal role (Shu et al., 2023). Sirtuin 1 (SIRT1), an NAD^+^-dependent deacetylase, has garnered significant attention as a central modulator of autophagy and cellular stress responses (Tang et al., 2024). SIRT1 is frequently upregulated in various human tumors, including HCC, and its inhibition has been shown to enhance the therapeutic efficacy of chemotherapeutic agents by impairing autophagic flux, particularly mitophagy (Zhang et al., 2025; Yao et al., 2022). Hence, SIRT1 may be an alternative HCC treatment target.

Berbamine (BBM) is a natural bis-benzylisoquinoline alkaloid derived from Berberis species, with documented anti-inflammatory, antibacterial, and broad anti-cancer properties (Zheng et al., 2025; Sadhu et al., 2024; Yan et al., 2025). It is already clinically approved for the treatment of leukopenia, indicating a favorable safety profile (Xiping et al., 2017). Previous studies have reported that BBM can suppress autophagy and sensitize human breast cancer and lung cancer cells to chemotherapy (Fu et al., 2018; Zhan et al., 2023). However, the precise mechanism underlying BBM-mediated autophagy inhibition, particularly its potential interplay with key regulatory nodes like SIRT1, remains largely unexplored. Critically, whether and how BBM modulates the SIRT1 pathway to inhibit autophagy and reverse chemoresistance in HCC is completely unknown. In this study, we therefore aimed to investigate whether BBM overcomes chemoresistance in HCC by inhibiting autophagy and to determine if this effect is mediated through the modulation of the SIRT1-TFEB axis. We initially evaluated the combination of BBM with 5-fluorouracil (5-FU), a chemotherapeutic agent widely used in HCC combination regimens in certain regions, and subsequently extended our investigation to sorafenib and paclitaxel to demonstrate broad-spectrum synergy.

## 2 Materials and methods

### 2.1 Reagents and antibodies

Berbamine (A0619) and Paclitaxel (A0177) were purchased from Chengdu Mansite BIO-Technology (Chengdu, China). 5-Fluorouracil (F100149) was obtained from Aladdin (Shanghai, China). Sorafenib (HY-10201), Baflomycin A1 (HY-100558), chloroquine (HY-17589A), rapamycin (AY-22989), resveratrol (HY-16561), and TFEB activator 1 (HY-135825) were purchased from MedChemExpress (Princeton, NJ, USA). Primary antibodies against β-actin (3700), p62 (5114), LC3B (3868), and Cathepsin B (31718) were from CST (Boston, MA, USA). Cathepsin D (AF1645) was purchased from Beyotime Biotechnology (Shanghai, China). Antibodies against TFEB (13372-1-AP), SIRT1 (13161-1-AP), Rab7 (55469-1-AP), and LAMP1 (67300-1-lg) were obtained from Proteintech (Wuhan, China). MT-ND4 (PA597298), MT-ND4L (PA5103953) were purchased from Thermo Fisher(MA, USA).

Peroxidase-labeled antibody to rabbit IgG (AS014), and peroxidase-labeled antibody to mouse IgG (AS003), FITC-conjugated goat anti-rabbit IgG (H + L) (AS011), Alexa Fluor 555-conjugated goat anti-mouse IgG (H + L) (AS057), Alexa Fluor 555-conjugated goat anti-rabbit IgG (H + L) (AS058) were purchased from ABclonal (Wuhan, China).

### 2.2 Cell culture

The Human hepatocellular carcinoma cell HepG2, SMMC-7721, as well as the mouse hepatocellular carcinoma cell line H22, were provided by the Laboratory of Molecular Oncology, Guangzhou University of Chinese Medicine, Guangzhou, China. All cell lines were maintained in Dulbecco’s modified Eagle’s medium (DMEM), supplemented with 10% (v/v) fetal bovine serum (FBS) and 1% penicillin/streptomycin, under a humidified atmosphere of 5% CO_2_ at 37 °C.

### 2.3 Cell viability assay

Cells were seeded in 96-well plates at a density of 4000 cells/well. After adherence, cells were treated with corresponding drugs for 24 or 48 h. Following this, 100 µL of a CCK-8 working solution (diluted 1:10 in phosphate-buffered saline, PBS) was introduced into each well, followed by a 2-h incubation at 37 °C. The OD value at 450 nm was measured by EnSpire® Multiscan Spectrum (PerkinElmer).

### 2.4 Colony formation assay

Cells (1 × 10^3^/well) were plated into 6-well plates. Incubation of the cells took 48 h with or without drugs, then changed of medium and allowed to grow until visible colonies formed (about 10-14 days). Cell colonies were fixed with 4% paraformaldehyde, stained with 0.1% crystal violet and then counted.

### 2.5 Annexin-V/PI apoptosis detection assay

Cells were seeded at a density of 2.0 × 10^6^ cells/well onto 6-well plates overnight and treated with drugs for 48 h. Following trypsinization, the cells were harvested and suspended in 600 µL 1 × binding buffer in FITC Annexin V Apoptosis Detection Kit (556547, BD). After that, 3 µL Annexin-V-FITC were added into per tube and the samples were incubated at 37 °C for 20 min without light, and then 5 µL/tube PI was added. Then, BD Accuri C6 flowcytometer was used to examine the single-cell staining for each sample.

### 2.6 Western blot

For Western blot, RIPA buffer (FD008, Fdbio science) and 1% PMSF (P0100, Solarbio) were used to lyse cultured cells. The total protein concentration was calculated based on the BCA protein assay (KGP903, KeyGen BioTECH) for each sample. Total protein (20 μg per lane) was separated on SDS–polyacrylamide gels and electrophoretically transferred to PVDF membranes (ISEQ00010, Millipore), then blocked with 5% skim milk in TBST solution for 4 h at room temperature. The membranes were incubated overnight at 4 °C with primary antibodies (1:1000) and then incubated with peroxidase-labeled anti-mouse IgG (AS004) or anti-rabbit IgG (AS014, 1:5000) for 1 h at room temperature. Enhanced chemiluminescence was used to detect protein bands (WBKLS, Millipore) and the signals were revealed by the Tanon GIS system. ImageJ software (NIH Image) was used to analyze the data. β-Actin was used as a loading control for normalization.

### 2.7 Immunofluorescence

The cells were seeded in 12-well plates with coverslips at 3.5 × 10^5^ cells per well. Cells were adhered to coverslips for 24 h, then treated with drugs 24 h and fixed with 4% paraformaldehyde for 20 min. Each coverslip was dropped with 300 µL of blocking solution (containing 0.3% Triton X-100, 10% FBS, 90% PBS) in the dark for 2 h. Subsequently, the slides were incubated at 4 °C overnight with primary antibody (diluted 1:200). Following PBS washes, the cells were incubated in the dark for 1 h with a fluorescent secondary antibody (diluted 1:200). The cells were stained with Hoechst 3342 (5 μg/mL) for 10 min, and then fixed with Prolong™ Diamond Antifade Mountant (P36965, Invitrogen). A confocal laser-scanning microscope (LSM 800; Carl Zeiss) was used to take images. Images were processed using ZEN 2 software (Carl Zeiss Inc., Jena, Germany). Quantification was performed using ImageJ software (NIH Image), with at least three randomly selected fields per sample.

### 2.8 LysoTracker staining

A mass of 1 × 10^5^ cells was seeded and incubated for 24 h in 15 mm confocal dishes. The cells were treated with relevant drugs for 24 h, then washed with PBS twice, stained in 75 nM LysoTracker Red (C1046, Beyontime) at 37 °C for 20 min under dark conditions. A confocal microscope was used to take images. Quantification was performed using ImageJ software (NIH Image), with at least three randomly selected fields per sample.

### 2.9 Intracellular NAD^+^/NADH assay

NAD^+^/NADH levels were measured with NAD^+^/NADH assay kit (S0175, Beyontime). Cells of HepG2, SMMC-7721 were seeded into 6-well plates at a density of 2.5 × 10^5^ cells per well overnight. Then fresh culture medium with varying concentrations of the drugs was replaced with the medium. After incubation for 24 h, the cells were washed and 200 μL of extracting solution was added. After centrifugation at low temperature, the supernatants were collected. It was strictly adhered to the instructions of the NAD^+^/NADH Assay Kit during all operations. Finally, the concentration of NAD^+^ or NADH was measure by a multifunctional microplate reader. The NAD^+^/NADH ratio was calculated as (NAD(H)total - NADH)/NADH.

### 2.10 Transcriptome sequencing assay

SMMC 7721 cells treated with 5 μM BBM for 24 h, and harvested in Trizol reagent (9109, Takara). The samples were sent to Sanshubio Co., Ltd. (Nantong, China) to performed transcriptome sequencing using the Illumina NovaSeq 6000 platform the standard procedures. Gene expression levels were quantified using HTSeq and normalized to Fragments Per Kilobase of transcript per Million mapped reads (FPKM). Differential expression analysis was conducted with the edgeR software package in R. Genes with an absolute log2 fold change (|log2FC|) > 1 and an adjusted p-value (padj) < 0.05 were considered statistically significantly differentially expressed. Functional enrichment analysis of these genes was performed based on the Reactome database.

### 2.11 Molecular docking analysis

The 3D Conformer of Berbamine (BBM) was downloaded from PubChem (Compound CID: 275182). The protein structures of AMPK, NRF-1 and ERRα were retrieved from RCSB PDB database. The structure of PGC-1α was obtained from AlphaFold Protein Structure Database. The water molecules and ligands in the protein-ligand complex structure were removed by using PyMOL. The AutoDock (version 4.2.6) was used to perform the molecular dockings and calculate the binding energies between BBM and each protein.

### 2.12 Animal experiments

In accordance with the guidelines of the Animal Care and Use Committee of Guangzhou University of Chinese Medicine, all animal studies were approved by the committee (No.20221230004). KunMing(KM) mice (male, 6-8 weeks old, 18-22g) were purchased from the Laboratory Animal Centre of Southern Medical University(Guangzhou, China), and housed in a standard laboratory environment with free access to water and food.

H22 cells (3.5 × 10^5^ cells in 200 µL PBS) were injected into the axilla of each KM mice. Daily measurements were performed after tumors were palpable. After that the tumor-bearing mice were randomly divided into four groups at random (n = 12). Mice in the treatment groups received intraperitoneal injections of BBM (50 mg/kg), 5-FU (5 mg/kg), or their combination every other day for 14 days, while the control group received an equal volume of vehicle. The anti-tumor treatment for tumor-bearing mice was initiated when the tumor volume (volume = length × width^2^/2) reached 0.1mm^3^. During the 14th day of administration, mice were sacrificed and tumor tissues were peeled off for further analysis.

### 2.13 Statistical analysis

Data visualization and statistical analysis were conducted using GraphPad Prism 8.0 (La Jolla, CA, USA). All experiments were performed with at least three independent biological replicates (n = 3). All quantitative data are expressed as the mean ± SD or SEM. Statistical significance was determined by one-way ANOVA followed by Tukey’s *post hoc* test (for multiple groups) or Student’s t-test (for two groups). The extent of fluorescent signal co-localization was determined by calculating Pearson’s correlation coefficient. In drug combination studies, synergy was assessed using the combination index (Q value) as per Jin et al. where Q = Eab/ (Ea + Eb - Ea × Eb) (Jin, 2004). Ea and Eb represent the efficacy of the individual agents, and Eab is the efficacy of the combination. The interaction was classified as synergistic if Q > 1.15, antagonistic if Q < 0.85, and additive for 0.85 ≤ Q ≤ 1.15. In all analyses, a p-value of less than 0.05 was considered statistically significant.

## 3 Results

### 3.1 BBM synergizes with 5-FU to suppress HCC

**FIGURE 1:**
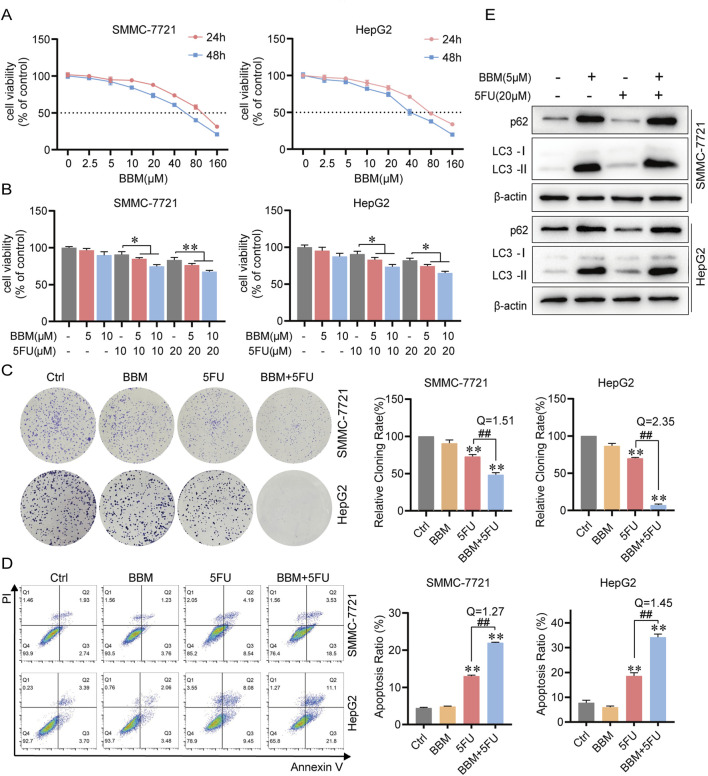
BBM synergizes with 5FU and inhibits autophagic flux in HCC cells. **(A)** Viability of SMMC-7721 and HepG2 cells treated with the indicated concentrations of BBM for 24 h or 48 h, measured by CCK-8 assay. **(B)** Viability of cells treated with BBM (5 or 10 μM) in combination with 5FU (10 or 20 μM) for 48 h. The Q value indicates a synergistic effect (Q > 1.15). **(C)** Representative images and quantification of colony formation assays in cells treated with BBM (5 μM) and/or 5FU (20 μM) for 48 h. **(D)** Apoptosis analysis by Annexin V/PI staining and flow cytometry in cells treated as in **(C)**. Representative flow cytometry plots are shown. **(E)** Western blot analysis of LC3 and p62 protein levels in cells treated with BBM (5 μM) and/or 5FU (20 μM) for 24 h. All values represent the mean ± SD (n = 3). *p < 0.05, **p < 0.01 vs. Ctrl. #*p* < 0.05, ##*p* < 0.01 vs. 5FU.

To evaluate the cytotoxicity of BBM, we treated the cells with increasing concentrations of BBM. CCK-8 assays revealed that BBM inhibited cell viability in a dose- and time-dependent manner, with IC50 values of 93.19 μM and 83.1 μM at 24 h, and 53.67 μM and 45.81 μM at 48 h for SMMC-7721 and HepG2 cells, respectively (Figure 1A). At concentrations ≤10 μM, BBM exhibited limited cytotoxicity, providing a safe window for combination therapy. We next investigated the potential synergistic effect between BBM and the chemotherapeutic agent 5-FU. Compared with the 5-FU monotherapy, the combination of BBM and 5-FU significantly reduced cell viability (Figure 1B), suppressed colony formation (Figure 1C), and induced apoptosis (Figure 1D). The calculated combination index (Q = 1.19-1.38 ≥ 1.15) confirmed a robust synergistic effect.

Given that autophagy modulation can influence chemosensitivity, we examined the expression of key autophagy markers. 5-FU treatment alone increased LC3-II levels and decreased p62, indicating autophagy induction. In contrast, the BBM and 5-FU combination led to the concurrent accumulation of both LC3-II and p62 (Figure 1E). p62 is primarily degraded through autophagy-dependent pathways, so p62 level is typically negatively associated with autophagy flux rate. This pattern suggests that while 5-FU activates autophagy, BBM blocks the autophagic flux without affecting the autophagy formation, preventing the degradation of autophagosomes and their cargo. This effect was consistent in both SMMC-7721 and HepG2 cells.

### 3.2 BBM enhances anti-tumor efficacy of 5-FU in HCC xenografts model

**FIGURE 2:**
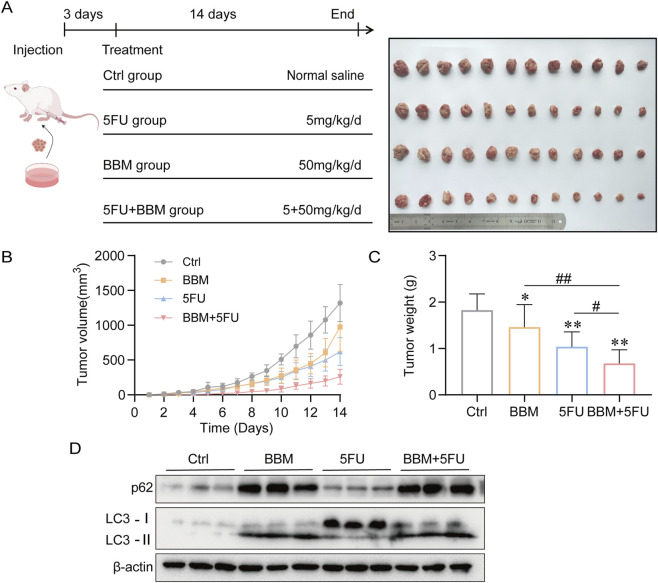
BBM enhances the anti-tumor efficacy of 5FU in a mouse xenograft model. **(A)** Schematic diagram of the *in vivo* experimental timeline. Tumor volume measurements of H22 xenograft-bearing mice treated with vehicle, BBM (50 mg/kg), 5FU (5 mg/kg), or their combination(n = 12 per group) over 14 days. Images of excised tumors from each treatment group at the endpoint. **(B)** Tumor growth curve. **(C)** Final tumor weight from each group. **(D)** Western blot analysis of LC3 and p62 protein levels in tumor lysates. Data are presented as mean ± SD. *p < 0.05, **p < 0.01 vs. control group; #*p* < 0.05, ##*p* < 0.01 vs. 5FU groups.

The synergistic effect observed *in vitro* was validated in an H22 xenograft mouse model. Treatment with BBM or 5-FU alone modestly inhibited tumor growth compared to the control. However, the combination of BBM and 5-FU resulted in a significant reduction in both tumor volume and tumor weight (the tumor weight inhibition rate of joint group was 60.0%, and that of BBM was 20.3%, 5-FU was 40.5%.), far exceeding the effects of either agent alone (Figures 2A–C). Western blot analysis of tumor tissues revealed that the combination therapy led to the accumulation of LC3-II and p62 (Figure 2D), mirroring our findings *in vitro* and confirming that BBM inhibits autophagic flux *in vivo*.

### 3.3 BBM blocks late-stage autophagic flux

#### 3.3.1 BBM induces autophagosome accumulation

**FIGURE 3:**
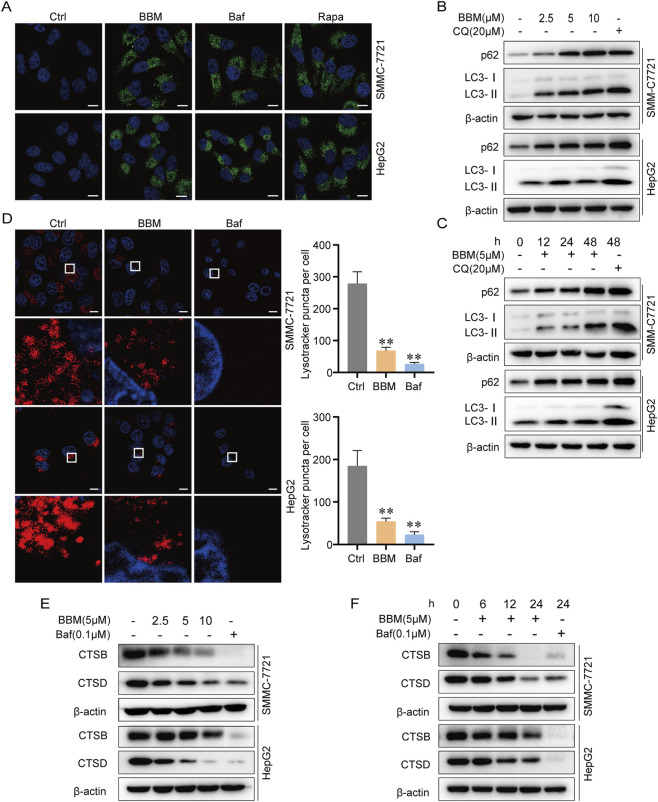
BBM inhibits the late stage of autophagic flux in HCC cells. **(A)** Representative confocal microscopy images of GFP-LC3 puncta in cells treated with BBM (5 μM), Baf (0.1 μM), or Rapa (1 μM) for 24 h. Scale bar, 10 μm. **(B,C)** BBM increases LC3B-II and p62 accumulation. Cells were exposed to various concentrations of BBM for 24 h or were treated with BBM for the durations indicated. Chloroquine (CQ, 20 µM) as positive control. **(D)** LysoTracker Red staining showing lysosomal acidification in cells treated with BBM (5 μM) or Baf (0.1 μM) for 24 h. Quantification of mean fluorescence intensity is shown on the right. Scale bar, 10 μm. **(E,F)** Western blot analysis of mature Cathepsin B (CTSB) and Cathepsin D (CTSD) levels in cells treated with BBM at indicated concentrations **(E)** or for indicated times **(F)**. Data are presented as mean ± SD from three independent biological replicates. *p < 0.05, **p < 0.01.

The accumulation of both LC3-II and p62 prompted us to investigate whether BBM impairs autophagy at late stage. Immunofluorescence analysis showed that BBM treatment significantly increased GFP-LC3 puncta, similar to the effects of the late-stage inhibitor Baf and the inducer Rapa (Figure 3A), indicating that BBM, like Baf, does not interfere with autophagosome formation. Western blot analysis further confirmed that BBM induced the accumulation of LC3-II and p62 in a concentration- and time-dependent manner, a phenotype shared with the inhibitor positive control CQ (Figures 3B,C), indicating that BBM, like CQ, acts at the late stage of autophagic flux.

#### 3.3.2 BBM inhibits lysosomal acidification

Lysosomal pH is a crucial determinant of its function. The V-ATPase, located on the lysosome membrane, actively transports hydrogen protons into the lysosome thereby enabling the maintenance of an acidic environment. We next investigated whether BBM also affects lysosomal function. LysoTracker staining, which labels acidic organelles, revealed that BBM treatment significantly reduced the fluorescence intensity, indicating impaired lysosomal acidity, similar to the V-ATPase inhibitor Baf (Figure 3D). Cathepsin B (CTSB) and cathepsin D (CTSD) are the primary lysosomal proteases involved in autophagy degradation. Consistent with impaired acidification, the levels of the lysosomal proteases CTSB and CTSD were suppressed by BBM in a concentration and time-dependent manner (Figures 3E,F). Our observations indicate that BBM exhibits a similar effect to Baf, suggesting its potential to inhibit lysosome acidification in HCC.

#### 3.3.3 BBM inhibits autophagosomal-lysosomal fusion

**FIGURE 4:**
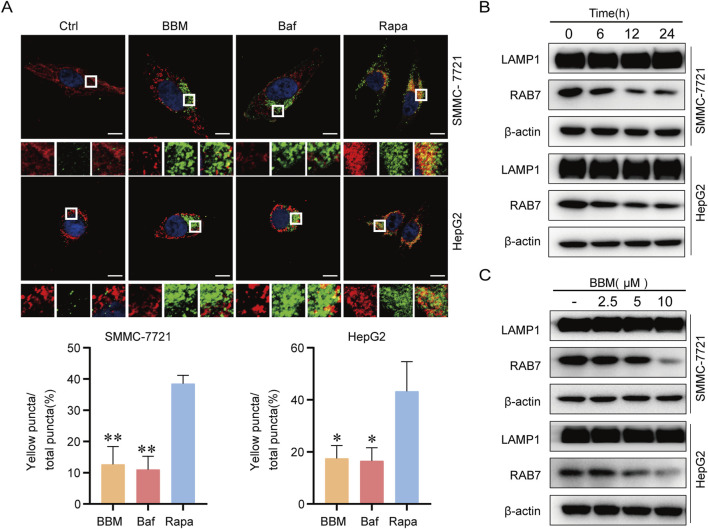
BBM blocks autophagosome-lysosome fusion. **(A)** Confocal microscopy analysis of autophagosome-lysosome fusion. Cells co-expressing mRFP-LC3 (red) and LAMP1-mGFP (green) were treated with BBM (5 μM), Baf (0.1 μM), or Rapa (1 μM) for 24 h. Yellow puncta indicate co-localization. Scale bar, 10 μm. **(B,C)** Western blot analysis of RAB7 and LAMP1 protein expression in cells treated with BBM at indicated times (B) or for indicated concentrations (C). Data are presented as mean ± SD from three independent biological replicates. *p < 0.05, **p < 0.01 vs. Rapa group.

The inhibition of late autophagy flux may also arise from the obstruction of the fusion process between autophagosomes and lysosomes. Lysosome associated membrane protein 1 (LAMP1), a primary constituent of the lysosomal membrane, assumes a pivotal role in facilitating autophagy-lysosome fusion. Using confocal microscopy, we found that BBM treatment drastically reduced the co-localization of mGFP-LC3 (autophagosomes) and LAMP1-mRFP (lysosomes), an effect comparable to Baf, in contrast to the autophagy activator Rapa which showed a large number of yellow spots of LAMP1 and LC3 fusion (Figure 4A). This demonstrates that BBM prevents the fusion of autophagosomes with lysosomes.

LAMP1 serves as a crucial marker for assessing the functionality of intracellular lysosomes. Notably, BBM did not affect the protein levels of LAMP1 (Figures 4B,C), suggesting the fusion defect is not due to a loss of these key lysosomal membrane proteins. Furthermore, BBM downregulated the level of RAB7 in a concentration- and time-dependent manner. RAB7 is a small GTPase critical for late endosomal/autophagosomal trafficking to lysosomes and their fusion (Figures 4B,C). These data indicate that BBM disrupts autophagy at multiple steps including autophagosomal trafficking and fusion with lysosomal.

### 3.4 BBM inhibits autophagy through SIRT1 acetylation

**FIGURE 5:**
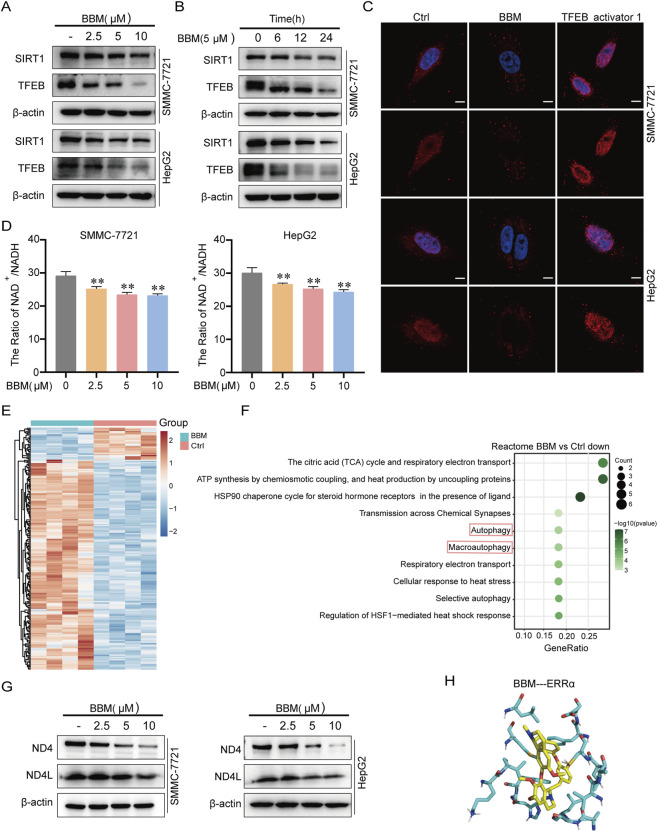
BBM suppresses the SIRT1-TFEB axis at the protein level. **(A,B)** Western blot analysis of SIRT1 and TFEB protein levels in cells treated with BBM at indicated concentrations **(A)** or for indicated times **(B)**. **(C)** Immunofluorescence analysis of TFEB subcellular localization. Cells were treated with BBM (5 μM) or TFEB activator 1 (0.5 μM) for 24 h and stained for TFEB (red) and DAPI (blue). Scale bar, 5 μm. **(D)** Intracellular NAD^+^ levels and NAD^+^/NADH ratio in cells treated with BBM (2.5, 5, 10 μM) for 24 h. All values represent the mean ± SD (n = 3), *p < 0.05, **p < 0.01. **(E)** Transcriptomic analysis of differentially expressed genes in SMMC-7721 cells after BBM (5 μM) treatment for 24 h. **(F)** Reactome pathway enrichment analysis of downregulated gene. **(G)** Western blot analysis of OXPHOS components (MT-ND4, MT-ND4L) in cells treated with BBM (0-10 μM) for 24 h. **(H)** Molecular docking analysis showing the predicted binding mode of BBM with ERRα. BBM is shown in green sticks, and hydrogen bonds are indicated as yellow dashed lines.

It was shown that BBM disrupts both autolysome fusion and degradation function, called the late stage of autophagy. Then we sought the upstream regulator. Acetylation is an important regulatory mechanism in autophagy. Sirtuin deacetylase 1 (SIRT1), a histone deacetylase reliant on nicotinamide adenine dinucleotide (NAD^+^), plays a role in modulating acetylation levels, participating in autophagy regulation, and activating various transcriptional factors, including TFEB. Western blot analysis showed that BBM treatment significantly downregulated both SIRT1 and TFEB protein levels in a concentration- and time-dependent manner (Figures 5A,B).

TFEB, a master regulator of lysosomal biogenesis and autophagy genes, are central to autophagy regulation. TFEB’s activity is dependent on its nuclear translocation. Immunofluorescence analysis revealed that with BBM treatment, the distribution of TFEB marked with red fluorescence in the cytoplasm was significantly reduced, while accumulated in the cell nucleus. BBM not only reduced total TFEB level but also potently inhibited its nuclear accumulation. In contrast, TFEB activator 1 (TFEBa), a specific TFEB agonist promoted robust nuclear localization (Figure 5C).

SIRT1, a NAD^+^-dependent deacetylase, utilizes NAD^+^ as its substrate, with its activity being contingent upon the cytoplasmic NAD^+^/NADH ratio. To investigate the impact of BBM on intracellular NAD^+^/NADH, we measured the cellular NAD^+^ and NADH levels. BBM treatment caused a significant decrease in the NAD^+^/NADH ratio (Figure 5D).

### 3.5 Transcriptomic analysis confirms post-transcriptional regulation of BBM

Previous experiments verified the regulation of BBM on autophagy at the protein level. Next, we used transcriptome sequencing to detect the effect of BBM at the mRNA level (Figure 5E). Through Reactome database enrichment analysis, BBM could downregulate autophagy-related pathway (Figure 5F) and upregulate transcription of HIC1,which is a transcriptional inhibitor of SIRT1. However, the mRNA levels of core autophagy and lysosomal genes, including SIRT1 and TFEB, were not significantly altered (Table 1). This crucial finding indicates that BBM regulates the SIRT1-TFEB axis and its downstream targets primarily at the post-transcriptional level or by affecting protein stability, rather than through transcriptional suppression. Besides, RNA-seq analysis confirmed that BBM treatment downregulated pathways related to respiratory electron transport (Figure 5F).

**TABLE 1:** Transcriptomic analysis of core autophagy and lysosomal genes in SMMC-7721 cells after BBM treatment.

Gene_name	BBM_FPKM	Ctrl_FPKM	log2FC	P value	Padj	Level
*LAMP1*	51.36	40.58	0.26	0.111	0.410	nosignificant
*RAB7A*	183.97	167.32	0.06	0.690	0.900	nosignificant
*SIRT1*	12.11	8.93	0.36	0.049	0.270	nosignificant
*TFEB*	8.79	8.21	0.02	0.917	1.000	nosignificant
*CTSD*	395.07	295.17	0.34	0.112	0.412	nosignificant
*MAP1LC3B*	50.92	40.46	0.25	0.109	0.407	nosignificant
*SQSTM1*	2996.77	2540.80	0.16	0.284	0.633	nosignificant
*CTSB*	232.51	197.77	0.16	0.288	0.638	nosignificant
*HIC1*	5.15	2.32	1.07	3.28559570540001e-09	7.19878780786049e-07	up

To gain deeper insight into the upstream mechanism by which BBM reduces the NAD^+^/NADH ratio and inhibits SIRT1, we performed additional analysis of our transcriptomic data. As shown in Table 2, BBM treatment for 24 h significantly downregulated nine genes, seven of which encode key components of the mitochondrial oxidative phosphorylation (OXPHOS) system, including MT-ND4L, MT-ND4, MT-ND5 (NADH dehydrogenase subunits), MT-CO1, MT-CO3 (cytochrome c oxidase subunits), and MT-ATP6, MT-ATP8 (ATP synthase subunits). All these genes were downregulated by more than two-fold (log2FC ≤ −1, padj <0.05). This coordinated suppression of OXPHOS genes provides a molecular explanation for the reduced NAD^+^/NADH ratio observed after BBM treatment (Figure 5D), as decreased expression of NADH dehydrogenase subunits would lower NAD^+^ regeneration. To validate the protein-level changes of OXPHOS components, we performed Western blot analysis for MT-ND4, MT-ND4L. As shown in Figure 5G, BBM treatment significantly reduced the protein expression of these OXPHOS subunits, consistent with the transcriptomic findings. Collectively, these results indicate that BBM suppresses OXPHOS gene and protein expression, leading to reduced NAD^+^ regeneration and subsequent SIRT1 inactivation.

**TABLE 2:** BBM downregulates mitochondrial OXPHOS genes at 24 h.

Gene_name	BBM_FPKM	Ctrl_FPKM	log2FC	Pvalue	Padj	Level
*MT-ND4L*	253.84	975.52	−1.92	9.94e-19	1.49e-14	down
*MT-ND4*	1637.62	5319.45	−1.65	3.50e-10	1.31e-06	down
*MT-CO1*	4901.91	11828.20	−1.23	6.74e-10	2.03e-06	down
*MT-ND5*	648.36	1589.12	−1.25	1.88e-08	4.04e-05	down
*MT-ATP8*	118.45	306.28	−1.33	2.62e-07	0.004	down
*XAF1*	7.81	16.72	−1.06	1.94e-05	0.014	down
*IFI44L*	11.90	24.36	−1.00	2.92e-05	0.018	down
*MT-ATP6*	2014.99	4604.93	−1.14	5.45e-05	0.025	down
*MT-CO3*	4440.18	9105.08	−0.98	8.65e-05	0.033	down

Differential expression analysis was performed using edgeR. Genes with |log2FC| ≥ 1 and padj <0.05 were considered significantly downregulated.

We next asked whether BBM directly interacts with upstream regulators of OXPHOS gene expression. Molecular docking analysis was performed using AutoDock 4.2.6. As summarized in Table 3, BBM exhibited favorable binding affinities with AMPK (−6.49 kcal/mol), PGC-1α (−7.31 kcal/mol), NRF-1 (−7.84 kcal/mol), and ERRα (−8.67 kcal/mol). Among these, ERRα showed the strongest binding energy, suggesting it may be a primary direct target of BBM. Given that ERRα is a master transcriptional regulator of OXPHOS genes, these data support a model in which BBM directly targets ERRα, leading to downregulation of OXPHOS gene expression, reduced NAD^+^/NADH ratio, and subsequent SIRT1 inactivation.

**TABLE 3:** Molecular docking analysis of BBM binding to energy-sensing proteins.

Protein	UniProtKB	PDB	Binding energy (kcal/mol)
AMPK	Q13131	6C9F	−6.49
PGC-1α	Q9UBK2	AlphaFold	−7.31
NRF-1	Q16656	8K4L	−7.84
ERRα	P11474	3K6P	−8.67

Molecular docking was performed using AutoDock 4.2.6. Binding energy (ΔG) is expressed in kcal/mol.

### 3.6 SIRT1 is the upstream regulator of BBM-induced autophagy blockade

**FIGURE 6:**
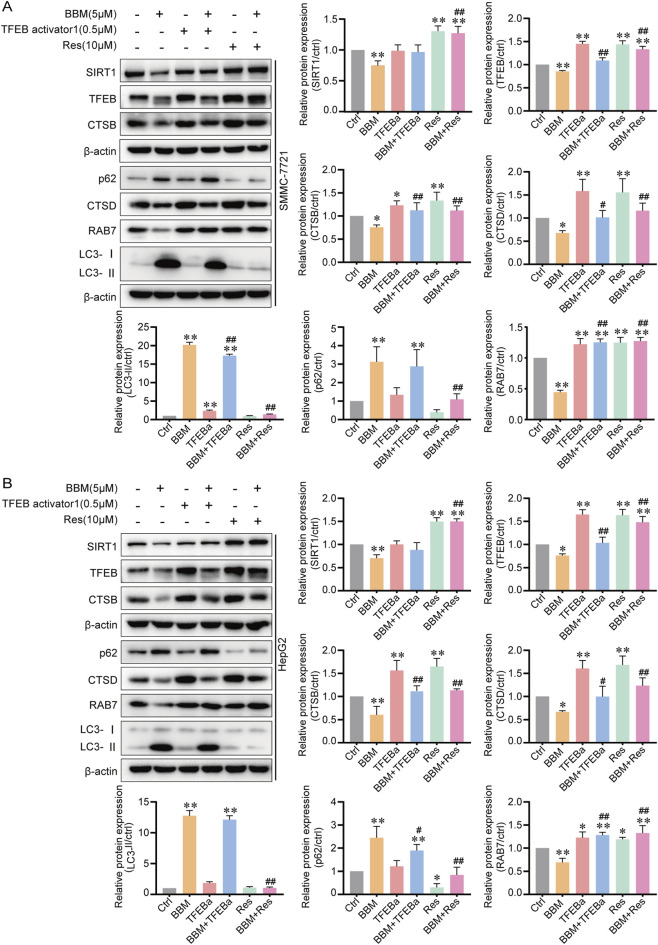
SIRT1 activation rescues BBM-induced autophagy blockade. **(A,B)** Western blot analysis of SIRT1, TFEB, lysosomal proteins (CTSB, CTSD, RAB7), and autophagy flux markers (p62, LC3) in cells treated with BBM (5 μM) in the presence or absence of the SIRT1 agonist Resveratrol (Res, 10 μM) or the TFEB agonist TFEB activator 1 (0.5 μM) for 24 h. Data are presented as mean ± SD (n = 3). *p < 0.05, **p < 0.01 vs. control; #*p* < 0.05, ##*p* < 0.01 vs. BBM-only group.

To establish the causal relationship among regulators, we performed rescue experiments by treating SMMC-7721 and HepG2 cells with BBM in the presence or absence of TFEB agonist (TFEBa) or SIRT1 agonist (Resveratrol, Res) (Figure 6). Compared with Ctrl, TFEBa upregulated the levels of TFEB, CTSB, CTSD and RAB7, but had no effect on p62 and SIRT1, indicating that overexpression of TFEB plays a regulatory role in CTSB, CTSD and RAB7. Compared with BBM alone, TFEBa partially reversed the inhibitory effects of BBM on TFEB, CTSB, CTSD and RAB7, but had little or no effect on SIRT1, p62 and LC3, indicating that BBM downregulated CTSB, CTSD and RAB7 through TFEB. However, TFEBa hardly reversed the inhibitory effect of BBM on autophagy. SIRT1 agonist Res significantly upregulated the levels of SIRT1, TFEB, CTSB, CTSD and RAB7, and downregulated of p62, compared with Ctrl, however, it has no effect on LC3-II and LC3-I level. This indicates that Res promotes the late autophagy flux but not the early stage including autophagy generation and the formation of autophagosomes. Compared with BBM alone, Res completely reversed the inhibitory effect of BBM on SIRT1, TFEB, CTSB, CTSD and RAB7 proteins, and weakened the upregulation of p62 and LC3-II by BBM. The results showed that SIRT1 plays a regulatory role in TFEB, CTSB, CTSD and RAB7, and BBM exerted the inhibitory impact on late-stage autophagy through SIRT1. The above results suggested that SIRT1 is the upstream regulator of TFEB, and TFEB is the upstream regulator of CTSB, CTSD and RAB7, both SIRT1 and TFEB can affect the fusion and acidification degradation of autophagy lysosomes.

### 3.7 BBM enhances chemosensitivity to multiple agents through autophagy inhibition

**FIGURE 7:**
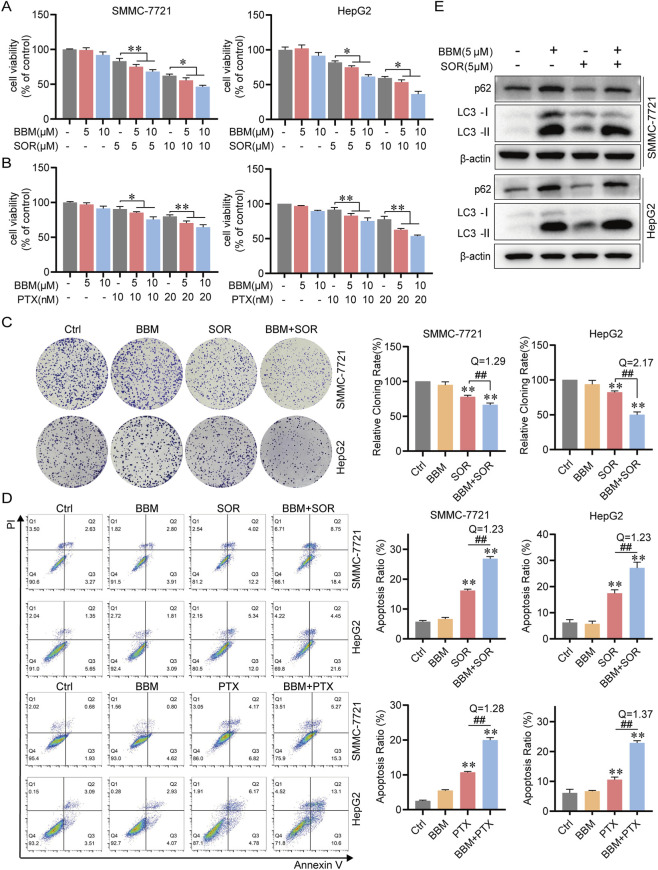
BBM sensitizes HCC cells to multiple chemotherapeutic agents. **(A)** Cell viability of SMMC-7721 and HepG2 cells treated with BBM (5 μM) in combination with Sorafenib (SOR, 10 μM) or Paclitaxel (PTX, 0.1 μM) for 48 h. **(B)** Representative images and quantification of colony formation assays in cells treated with BBM and/or SOR. **(C)** Apoptosis analysis by Annexin V/PI staining in cells treated with BBM and/or SOR/PTX. **(D)** Western blot analysis of LC3 and p62 levels in cells treated with BBM and/or SOR. Data are presented as mean ± SD (n = 3). *p < 0.05, **p < 0.01 vs. control; #*p* < 0.05, ##*p* < 0.01 vs. single-agent groups.

Regarding the choice of combination partners, we initially evaluated BBM with 5-FU because 5-FU remains a commonly used chemotherapeutic agent in HCC, particularly in combination regimens in certain clinical settings. We then extended our analysis to sorafenib (SOR) and paclitaxel (PTX). BBM synergized (Q > 1.15) with other chemotherapeutic agents, including SOR and PTX, to inhibit cell viability (Figures 7A,B) and colony formation (Figure 7C), and to induce apoptosis (Figure 7D). SOR treatment alone decreased p62, indicating flux activation, but this was reversed by co-treatment with BBM, which caused pronounced accumulation of both LC3-II and p62 (Figure 7E). This confirms that the chemosensitizing effect of BBM is generalizable and is mediated through its potent inhibition of autophagic flux, demonstrating that BBM synergizes with multiple agents with distinct mechanisms of action. This broad-spectrum synergy suggests that BBM’s autophagy-inhibitory effect is generalizable and may be applicable to various chemotherapeutic regimens.

## 4 Discussion

Chemoresistance driven by pro-survival autophagy remains a major therapeutic challenge in advanced HCC. In this study, we demonstrated that BBM, a naturally occurring alkaloid, functions as a novel autophagy inhibitor that potently sensitizes HCC cells to multiple chemotherapeutic agents both *in vitro* and *in vivo*. Autophagy is a dynamic, multi-step process. We found that BBM induces autophagosome accumulation and blocks autophagic flux in HCC cells. We provide compelling evidence that BBM not only inhibits the fusion of autophagosomes with lysosomes but also impairs lysosomal acidification, thereby affecting the degradation within autolysosomes, without compromising autophagosome formation and lysosomal structural integrity. We further elucidated that the core mechanism involves BBM-mediated suppression of SIRT1 expression and activity.

The fusion of autophagosomes and lysosomes is a critical step in autophagy, mediated by the SNARE complex. The canonical STX17-SNAP29-VAMP8 complex drives this process, and disruption of any component impairs fusion, causing autophagosome accumulation (Tian et al., 2020; Tian et al., 2021). Acetylation serves as a pivotal regulatory mechanism targeting multiple SNARE proteins. For instance, deacetylation of STX17, mediated by deacetylases such as HDAC2, promotes its interaction with SNAP29 and formation of SNARE complex (Jian et al., 2025; Shen et al., 2021). Beyond STX17, SNAP47 has been identified as another key target. Its acetylation status orchestrated directly controls HOPS complex recruitment and SNARE assembly, deacetylation promotes while acetylation inhibits. Our immunofluorescence experiments confirmed that BBM inhibits this fusion process in HCC cells. Crucially, the reversal of BBM’s autophagic inhibition upon SIRT1 agonism suggests that SIRT1 may influence the SNARE complex, although this requires further experimental validation. We did not detect the deacetylation of SNARE complex component, but we found RAB7 inhibited by BBM. RAB7 is a small GTPase vital for endosomal-lysosomal trafficking and autophagosome-lysosome fusion (Xiao et al., 2021; Boda et al., 2025), and influenced by SIRT1 (Zhu et al., 2023). Inhibiting SIRT1 has been shown to diminish RAB7 protein levels and hinder autolysosome formation (Luchetti et al., 2022). We found the inhibitory effects of BBM on RAB7 and autophagic flux were reversed by SIRT1 agonist. All the above suggested that BBM repress the fusion of autophagosomes and lysosomes through SIRT1-RAB7.

Lysosomal acidification is another pivotal process affecting autophagic flux, and its attenuation can lead to flux defects (Senturk et al., 2019). The activity of lysosomal hydrolases, such as CTSB and CTSD, depends on the acidic pH maintained by the proton pump V-ATPase. BBM treatment concentration- and time-dependently inhibited the expression of mature CTSB and CTSD, consistent with its role in suppressing lysosomal acidification, as evidenced by our LysoTracker results above.

Post-translational modifications, including phosphorylation, ubiquitination, and acetylation, play crucial roles in autophagy regulation (Xu and Wan, 2023). Acetylation exerts its regulation of autophagy at the transcriptional level through its influence on the epigenetic landscape (histones) and the key transcription factor TFEB. TFEB is a master regulator of lysosomal biogenesis and autophagy, controlling genes involved in autophagosome formation, fusion, and substrate degradation, that is to say, the entire process of autophagy (Settembre et al., 2011). Its activity is tightly linked to its subcellular localization (Wu et al., 2019). TFEB shuttles into the nucleus in response to various stresses, where it can be deacetylated by SIRT1 to activate the transcription of lysosomal and autophagy genes (Kory et al., 2020). TFEB is regulated by SIRT1-mediated deacetylation at K116, which promotes lysosomal biogenesis (Hariharan et al., 2010). We found that BBM significantly downregulated the levels of both SIRT1 and TFEB in HCC cells and impeded TFEB nuclear translocation, and then weakened the levels of RAB7, CTSB and CTSD which related to autolysosomal fusion and acidification.

SIRT1 deacetylates numerous autophagy-related proteins to participate in autophagy regulation. Our key question was whether BBM’s downregulation of TFEB, CTSB, CTSD, and RAB7 was mediated through SIRT1. Using agonists for rescue experiments, we found that TFEB overexpression by TFEBa only partially reversed BBM’s effects on CTSB, CTSD, and RAB7 but could not rescue SIRT1, the p62 accumulation and the blocked autophagic flux. This indicates that inhibiting TFEB affects lysosomal proteases and RAB7, but restoring TFEB activity did not reverse BBM’s concurrent blockade autophagic flux when SIRT1 was still inhibited. In stark contrast, SIRT1 agonist completely reversed BBM’s effects on SIRT1 itself, TFEB, CTSB, CTSD, RAB7, p62, and LC3II restoring autophagic flux. It unequivocally identifies that (1) SIRT1 is the primary upstream target of BBM. TFEB acts downstream of SIRT1 and upstream of CTSB, CTSD, RAB7 in mediating its effects on autophagy; (2) SIRT1 adjusts other key effectors, potentially including SNARE complex proteins, through which BBM inhibits autophagy not relying on TFEB.

Our findings that BBM inhibits autophagy through SIRT1 and subsequently TFEB function are strongly supported by a contemporaneous study which mechanistically delineated how enhanced SIRT1-TFEB interaction directly reduces TFEB acetylation to promote lysosome biogenesis and autophagic flux (Xu et al., 2025). Their work identified the functional consequences of SIRT1-mediated TFEB deacetylation. This convergence of evidence from different biological contexts (mycotoxin toxicity and cancer chemotherapy) highlights the fundamental nature of the SIRT1-TFEB axis in autophagy regulation. Thus, as we proposed, BBM represses the late stage of autophagic flux by SIRT1-TFEB axis and SIRT1-SNARE.

SIRT1 activity is dependent on the cytosolic NAD^+^/NADH ratio (Zhang et al., 2019). NAD^+^ is an essential substrate for sirtuins and other enzymes mediating post-translational modifications (Cambronne et al., 2016). Fluctuations in intracellular NAD^+^ concentration can modulate SIRT1 activity. NAD^+^ and SIRT1 can also modulate the activity of lysosomal cathepsins, increased NAD^+^ and SIRT1 expression upregulates mature CTSD levels, enhancing lysosomal function (Dong et al., 2021). We found that BBM treatment reduced the NAD^+^/NADH ratio in HCC cells, which we postulate is a key factor in the downregulation of SIRT1 by BBM.

Our transcriptomic analysis revealed that BBM markedly downregulates multiple OXPHOS genes, particularly those encoding NADH dehydrogenase (complex I) and ATP synthase (complex V) subunits (Table 2). This transcriptional signature is highly consistent with inhibition of ERRα, a nuclear receptor that serves as a master regulator of OXPHOS gene expression. ERRα regulates genes involved in mitochondrial biogenesis and oxidative metabolism (Vanacker and Forcet, 2024). The observation that BBM downregulates ERRα target genes without significantly altering ERRα mRNA levels suggests a post-transcriptional or direct inhibitory mechanism.

Molecular docking predicted that BBM binds to ERRα with a favorable affinity (−8.67 kcal/mol), comparable to known ERRα modulators. Moreover, preliminary cellular thermal shift assay (CETSA) data suggest that BBM may directly engage with ERRα in living cells; complete results will be reported in a future study. We therefore propose that BBM directly binds to ERRα, thereby inhibiting its activity. This inhibition leads to decreased expression of OXPHOS genes, which in turn reduces NAD^+^ regeneration (since NADH dehydrogenase is a major consumer of NADH and regenerator of NAD^+^). The consequent decline in the NAD^+^/NADH ratio (Figure 5D) impairs SIRT1 deacetylase activity because NAD^+^ is an obligate co-substrate for SIRT1.

Notably, the protein level of SIRT1 was also significantly reduced by BBM (Figures 5A,B), whereas its mRNA level remained unchanged (Table 1). This discrepancy suggests that BBM promotes SIRT1 protein degradation, possibly because NAD^+^ depletion destabilizes the SIRT1 protein conformation. Given that NAD^+^ binding is critical for maintaining the catalytically competent conformation of SIRT1 (Davenport et al., 2014), it is plausible that prolonged NAD^+^ depletion may destabilize the SIRT1 protein, potentially making it more susceptible to proteasomal degradation. This could explain the discrepancy between unchanged SIRT1 mRNA levels and reduced protein levels upon BBM treatment. Thus, BBM imposes a dual hit on SIRT1: (i) direct inhibition of its enzymatic activity due to NAD^+^ shortage, and (ii) reduction of its protein levels *via* destabilization. This dual suppression amplifies the blockade of SIRT1-dependent autophagic processes, including TFEB nuclear translocation and the expression/activity of RAB7, CTSB, and CTSD.

In addition to ERRα, BBM also showed moderate binding affinity to AMPK, PGC-1α, and NRF-1 (Table 3). These proteins form an interconnected energy-sensing network: AMPK senses AMP/ATP ratio, activates PGC-1α, and together with NRF-1 and ERRα coordinates OXPHOS gene expression (Fan et al., 2025; Herzig and Shaw, 2018; Scarpulla, 2011). Although further studies are needed to determine the relative contribution of each potential target, our data suggest that BBM may exert pleiotropic effects on this network, collectively suppressing mitochondrial respiration and NAD^+^ regeneration.

Transcriptome sequencing revealed that BBM downregulated autophagy-related pathways but did not significantly alter the transcription of the specific autophagy-related proteins we investigated, supporting the conclusion that BBM primarily regulates these proteins at the post-transcriptional or post-translational level. The result is also consistent with the conclusion that SIRT1 regulates its downstream targets at the translational level through deacetylation. Intriguingly, BBM upregulated the transcription of HIC1, a known transcriptional repressor of SIRT1. This suggests a potential transcriptional mechanism contributing to the observed suppression of SIRT1 protein levels, alongside the post-translational effects. Separately, the transcriptome data also indicated that BBM suppressed pathways related to mitochondrial respiratory electron transport. This finding is consistent with our observed decrease in the NAD^+^/NADH ratio, as impaired mitochondrial function can lead to a reduction in this ratio. The diminished NAD^+^/NADH ratio likely further compromises the activity of the NAD^+^-dependent deacetylase SIRT1. These transcriptional changes (HIC1 upregulation and respiration suppression) potentially work in concert to inhibit SIRT1 at both the expression and activity levels, ultimately influencing downstream deacetylation events and autophagy.

High chemoresistance severely limits the efficacy of chemotherapy. Sorafenib, for instance, only extends the median overall survival of advanced HCC patients by 2-3 months (Adler and Sutcliffe, 2017; Meyer, 2018). Autophagy is a recognized contributor to chemoresistance. Combining chemotherapy with autophagy inhibitors, such as the late-stage inhibitors CQ and HCQ, is a promising strategy (Compter et al., 2021; Haas et al., 2019). However, the high doses of CQ required for antitumor efficacy can lead to significant toxic side effects (Melles et al., 2023). In contrast, BBM, already approved for clinical use in leukopenia, offers a favorable safety profile. Our study validates the chemosensitizing effect of BBM in HCC and identifies a previously unreported mechanism-autophagy inhibition *via* the SIRT1-TFEB axis. Beyond conventional chemotherapy, the potential combination of BBM with current first-line standard-of-care regimens for advanced HCC, including immune checkpoint inhibitors (ICIs) and next-generation tyrosine kinase inhibitors (TKIs) such as lenvatinib, deserves consideration. Given that autophagy promotes resistance to both ICIs and TKIs (Kabir et al., 2025; Gao et al., 2022), BBM’s ability to block late-stage autophagic flux may sensitize HCC cells to these agents. This hypothesis warrants future investigation in appropriate preclinical models and clinical trials. The favorable safety profile of BBM (already approved for leukopenia) further supports its potential as an adjunctive agent in combination immunotherapy or targeted therapy.

A limitation of our study is that the direct molecular target of BBM upstream of SIRT1 remains to be identified. Furthermore, the specific SIRT1 substrate(s) responsible for the TFEB-independent inhibition of autophagic flux have yet to be determined. Future studies employing techniques like drug affinity responsive target stability (DARTS) or chemical proteomics could help unveil the primary binding partner of BBM.

## 5 Conclusion

SIRT1 is a key regulator of autophagy. SIRT1 modulates proteins such as TFEB through deacetylation, further regulating critical proteins involved in autophagosome-lysosome fusion, acidification and degradation, including CTSB, CTSD, and RAB7, thereby promoting autophagic flux. BBM downregulates the transcription and protein expression of NADH dehydrogenases, leading to reduced NAD^+^ regeneration and subsequent inhibition of SIRT1 activity. Consequently, by inhibiting SIRT1, BBM suppresses not only the fusion of autolysosomes but also acidification and substrate degradation, thereby blocking the late-stage autophagic flux in liver cancer cells and enhancing the efficacy of chemotherapy drugs like 5-FU *in vivo* and *in vitro*.

## Data Availability

The datasets presented in this study can be found in online repositories. The names of the repository/repositories and accession number(s) can be found in the article/Supplementary Material.
