# Supplementary material for: Berbamine sensitizes hepatocellular carcinoma to chemotherapy by inhibiting autophagy via modulating SIRT1-mediated acetylation
Source: Front Pharmacol. 2026 May 14;17:1763828. doi: 10.3389/fphar.2026.1763828 (PMC13216193; doi:10.3389/fphar.2026.1763828)

Applied Biosystems
GeneMapper ID v3.2

20240527

Sample File	Sample Name	Panel	SQO	OS	SQ
32_H04_Cellidentification-1--0528.fsa	hepG2	21Plex_STR_Panel_v1.2			

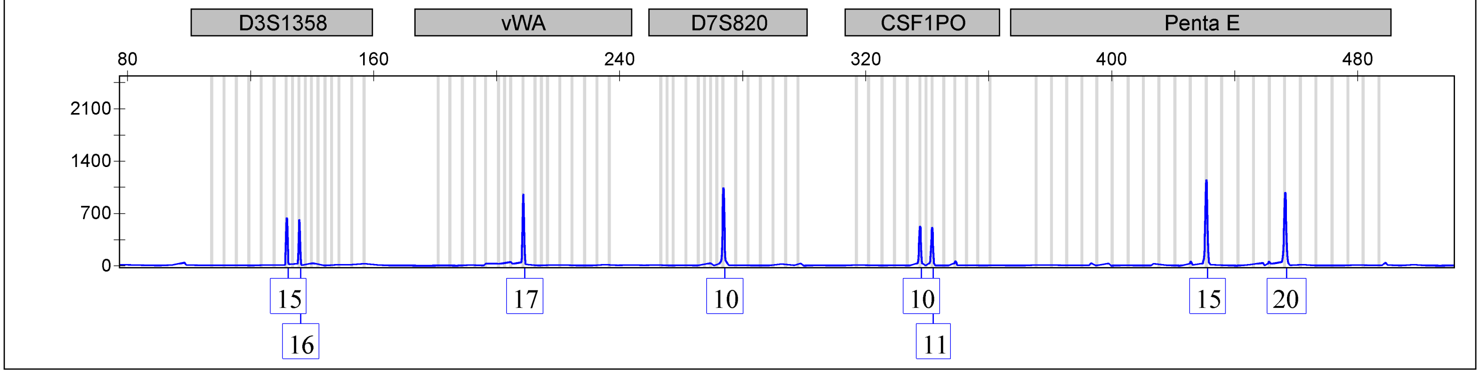

32_H04_Cellidentification-1--0528.fsa	hepG2	21Plex_STR_Panel_v1.2			

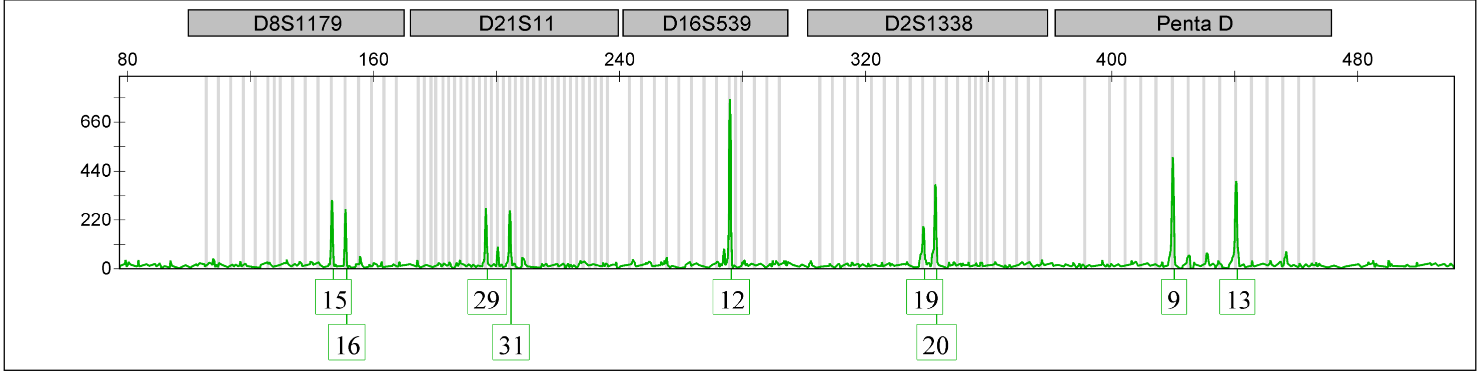

32_H04_Cellidentification-1--0528.fsa	hepG2	21Plex_STR_Panel_v1.2			

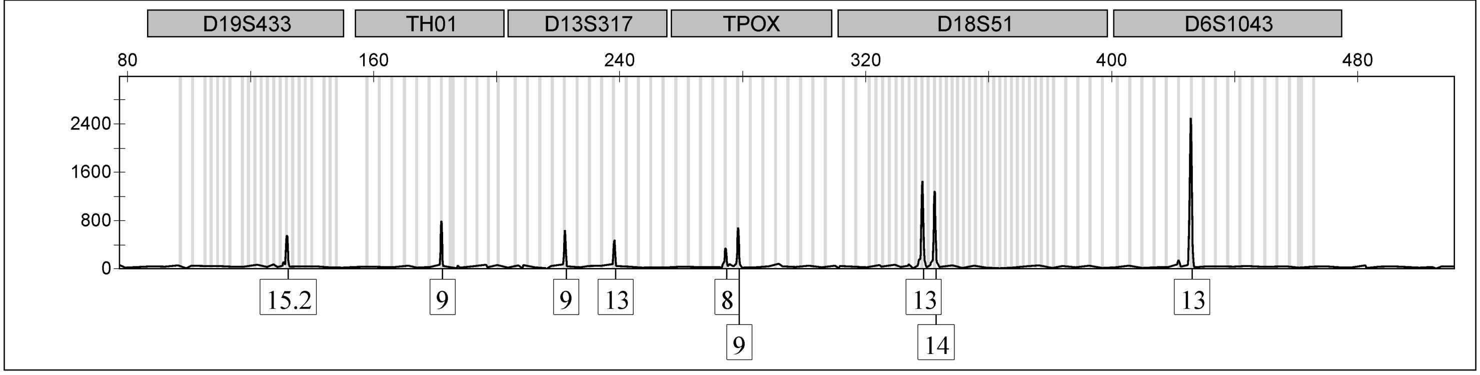

32_H04_Cellidentification-1--0528.fsa	hepG2	21Plex_STR_Panel_v1.2			

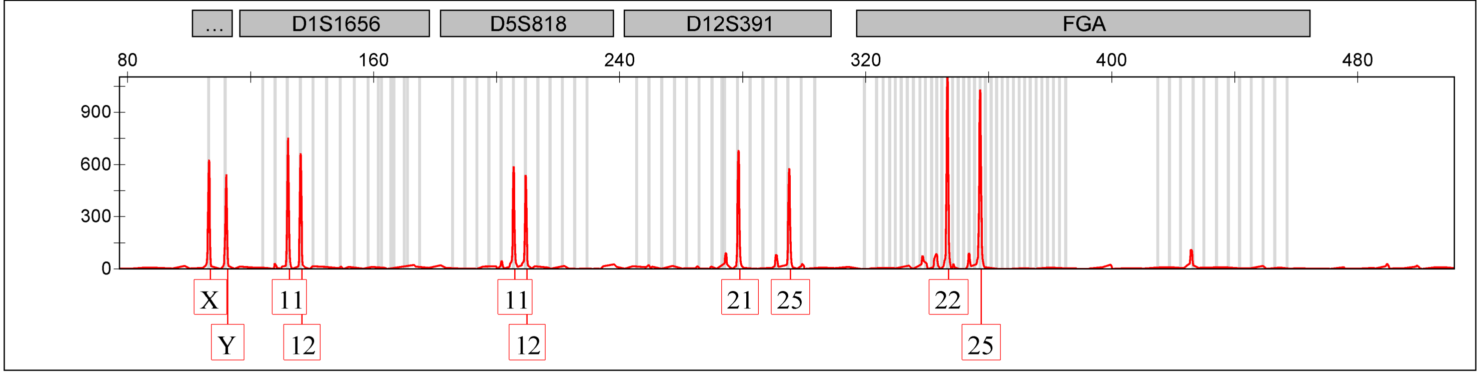

Applied Biosystems

GeneMapper ID v3.2

20240527

Sample File	Sample Name	Panel	SQO	OS	SQ
32_H04_Cellidentification-1--0528.fsa	hepG2	21Plex_STR_Panel_v1.2			

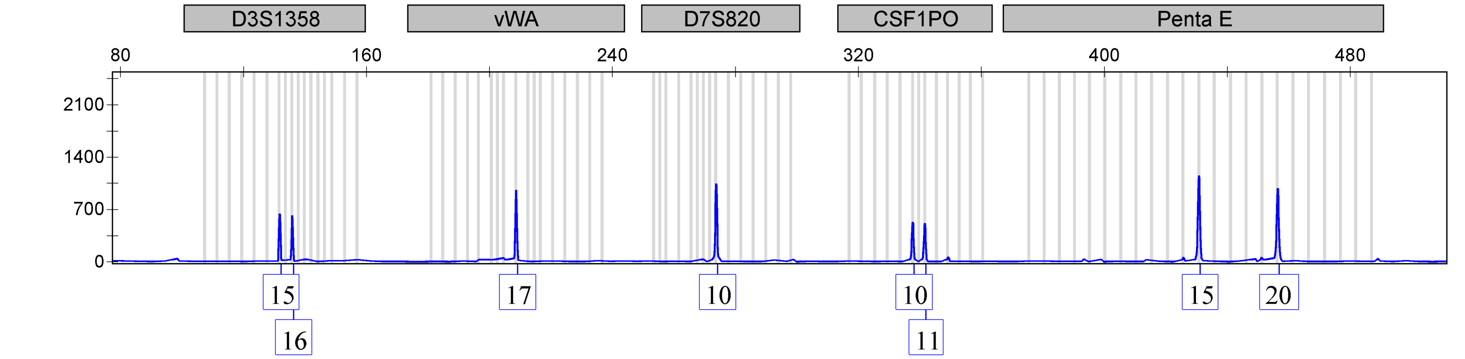

Sample File	Sample Name	Panel	SQO	OS	SQ
32_H04_Cellidentification-1--0528.fsa	hepG2	21Plex_STR_Panel_v1.2			

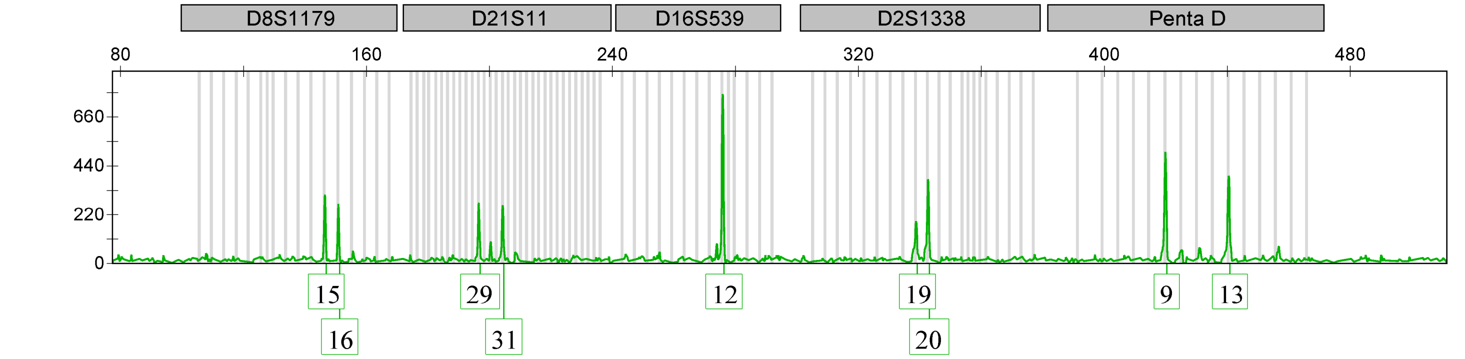

Sample File	Sample Name	Panel	SQO	OS	SQ
32_H04_Cellidentification-1--0528.fsa	hepG2	21Plex_STR_Panel_v1.2			

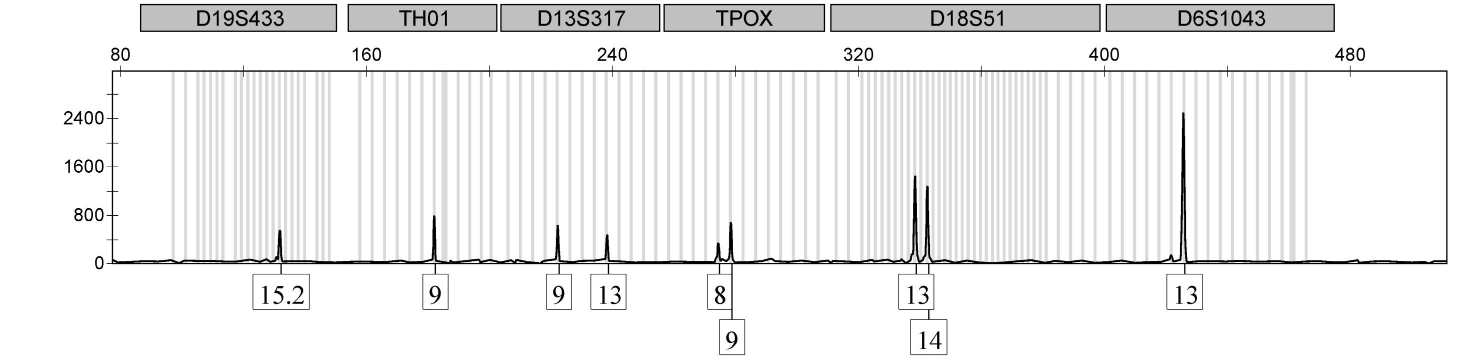

Sample File	Sample Name	Panel	SQO	OS	SQ
32_H04_Cellidentification-1--0528.fsa	hepG2	21Plex_STR_Panel_v1.2			

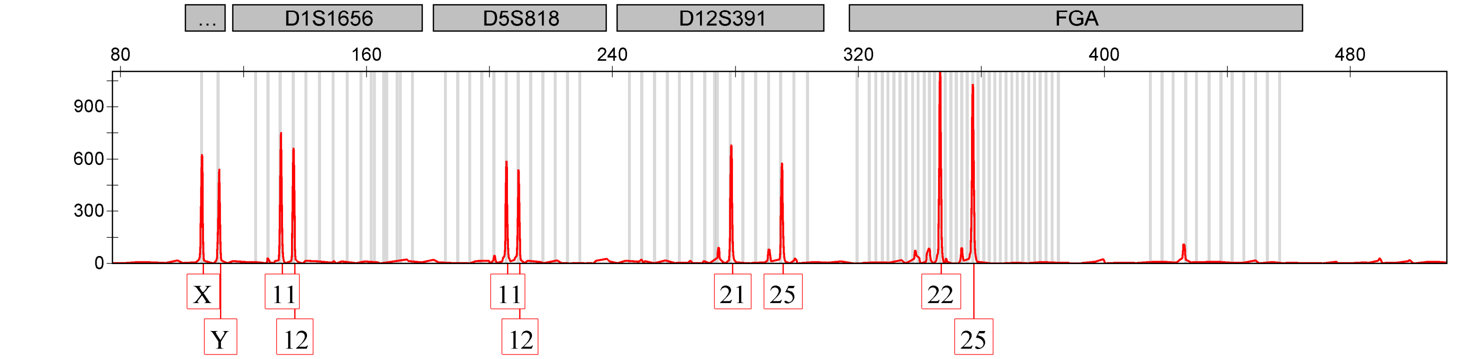

Supplement: Supplementary file 1 [file DataSheet1.pdf]
