# Supplementary figures and images for: Berbamine sensitizes hepatocellular carcinoma to chemotherapy by inhibiting autophagy via modulating SIRT1-mediated acetylation
Source: Front Pharmacol. 2026 May 14;17:1763828. doi: 10.3389/fphar.2026.1763828 (PMC13216193; doi:10.3389/fphar.2026.1763828)

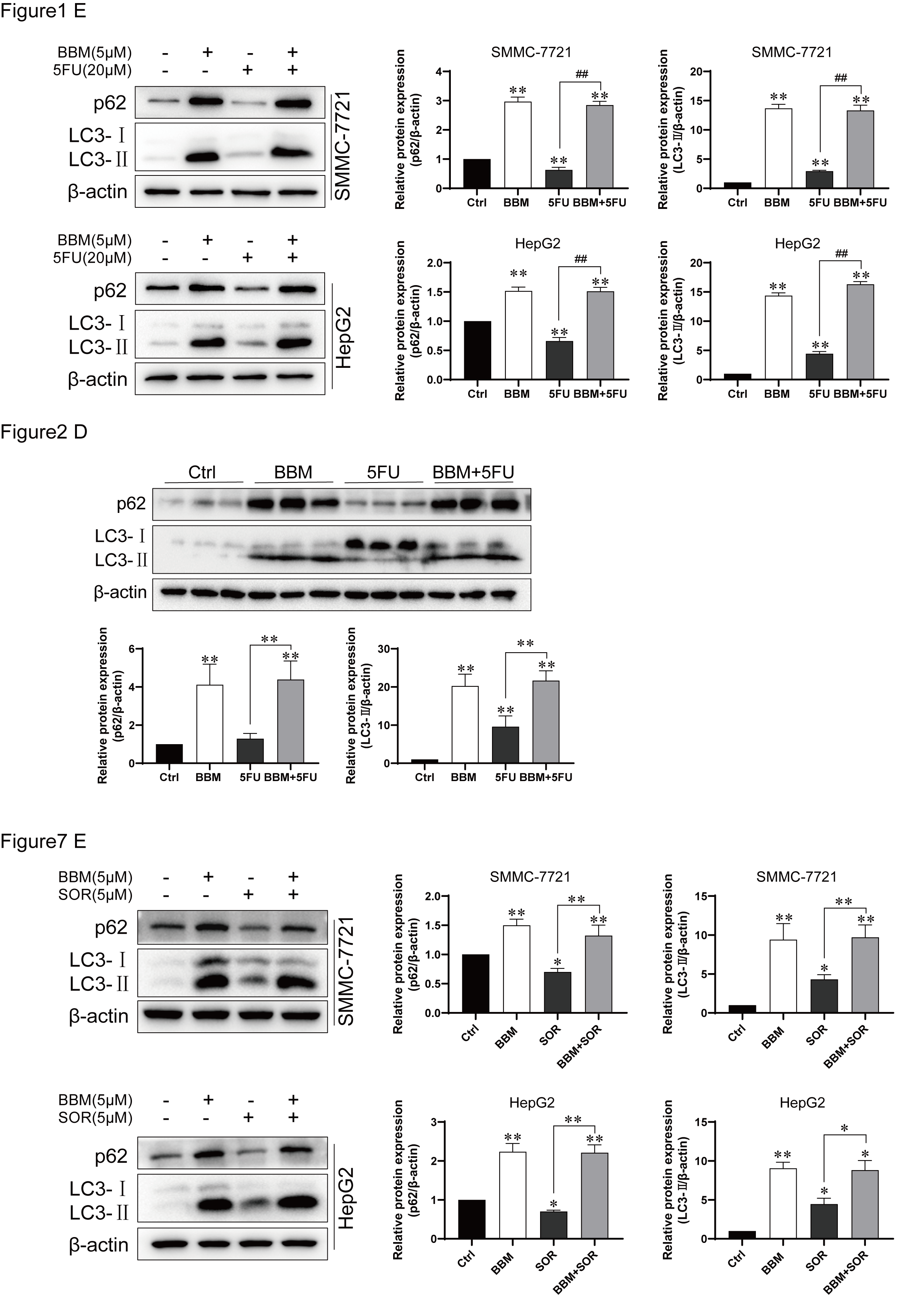

Supplement: Supplementary file 2 [file Image2.png]

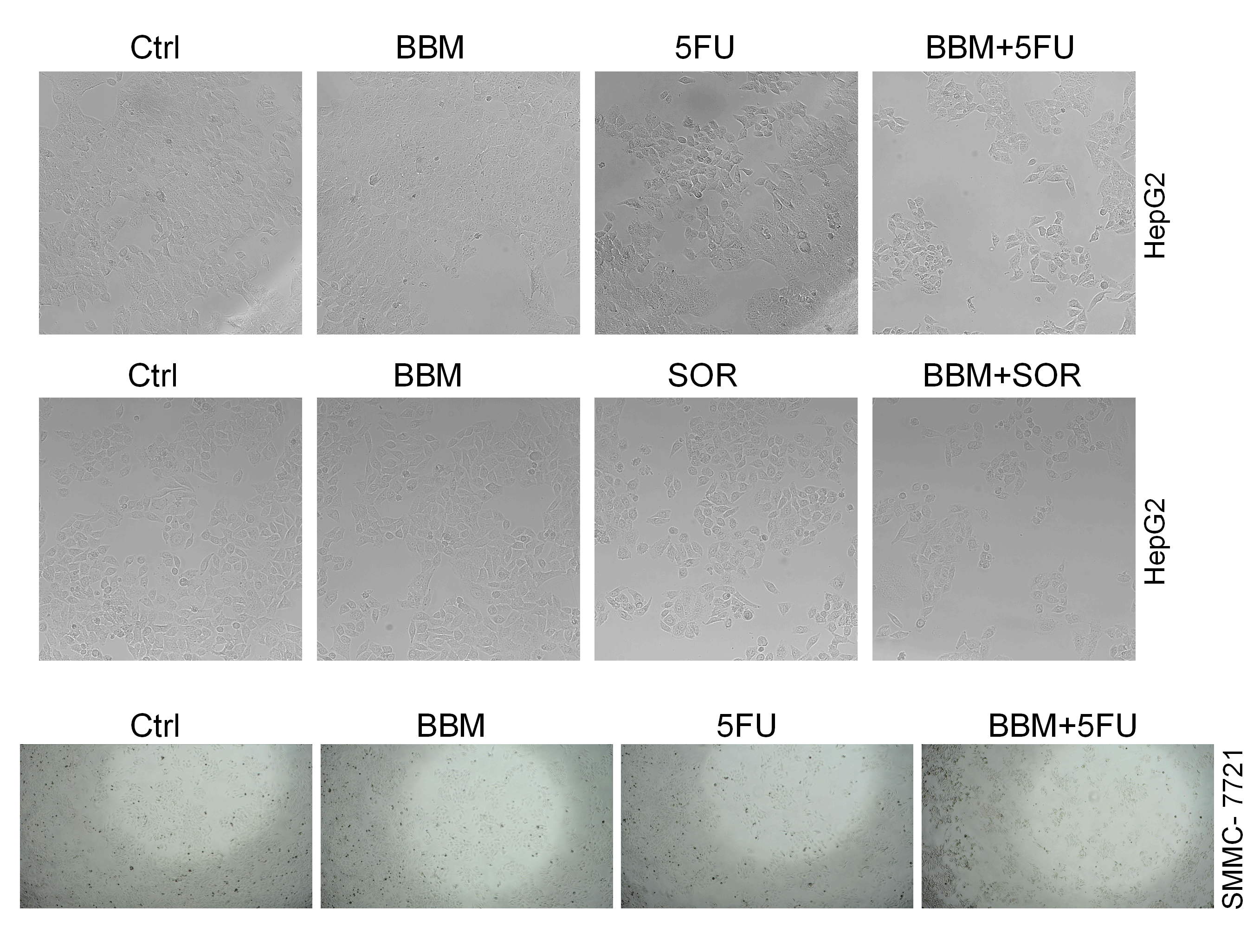

Supplement: Supplementary file 3 [file Image1.png]
